# Consumer loneliness: A systematic review and research agenda

**DOI:** 10.3389/fpsyg.2023.1071341

**Published:** 2023-01-20

**Authors:** Shanshan Huang, Mingfei Li

**Affiliations:** School of Economics and Management, Hubei Minzu University, Enshi, Hubei, China

**Keywords:** consumer loneliness, consumer behaviors, empirical research, quality of life, lonely consumers

## Abstract

Treading on the heels of the spread of the coronavirus, the “loneliness virus” has been capturing territories globally. Consumers are not immune to loneliness. Although academics and the general public have recognized the devastating effects of loneliness, the academic attention given to consumer loneliness (CL) is scattered and fragmentary. The purpose of this article is to systematically review the antecedents (predictors and alleviators) and consequences (consumer behaviors, emotions, preferences, attitudes, and cognition) of CL in various consumption contexts. This review also presents findings on CL as a mediator and moderator in consumer studies. This work adds to the growing body of CL literature by synthesizing the existing findings and knowledge. More importantly, we present a future research agenda by linking CL to significant research lines and detailed implications for practitioners in the marketplace.

## 1. Introduction

Loneliness was a global issue even before the outbreak of the COVID-19 pandemic (Fumagalli et al., 2022). People in modern societies worldwide have explicitly reported their loneliness, which is increasingly prevalent (Pieters, 2013; Kim and Jang, 2017; Wang et al., 2021). Loneliness has been acknowledged as a phenomenon resulting from global societal shifts and changing demographics, including reduced family sizes, fragmented family structures, increased relocation frequencies, growing metropolitan cities, more technology use, and aging populations (Mittal and Silvera, 2018). Prior research has related loneliness to a range of negative health outcomes (heart disease, suicide, mortality, etc.) and psychological outcomes (depression, anxiety, anger, etc.) across life spans and cultures (Heinrich and Gullone, 2006; Cacioppo and Cacioppo, 2018; Chen et al., 2021). Therefore, loneliness has been viewed as “part and parcel of human” and needs to be alleviated for physical and psychological functioning (Hawkley and Cacioppo, 2010).

Consumers are not immune to loneliness (Rippé et al., 2018; Dalman et al., 2021). Increasing research attention has been paid to the phenomenon of consumer loneliness (CL) in various contexts (Pieters, 2013; Pittman and Reich, 2016; Tan and Lu, 2019; Odekerken-Schröder et al., 2020; Loh et al., 2021). Despite the proliferation of research on CL, the literature is neither coherent nor explanatory and has provided an inadequate understanding of CL. Furthermore, little research has investigated the main issues and provided a holistic view of the research evidence to understand CL.

This review makes four major contributions. First, to the best of our knowledge, this study provides the first overview of CL research. We develop a systematic understanding of CL by integrating the extant knowledge of the CL phenomenon. This study clarifies the content, boundaries, and core subjects of CL studies. Second, this paper presents an in-depth examination of the role of CLs in consumer research and sheds light on empirical studies by identifying the antecedents and consequences of CL and its role as a mediating and moderating variable. Third, this review provides a theoretical basis for the future development of CL research. Based on the current state of knowledge, this study presents a roadmap for future CL research by identifying critical gaps and provides a unique perspective that enriches and broadens consumer research. Finally, this study goes beyond synthesizing existing research by elaborating on several practical implications that constitute a basis for future marketing programs.

## 2. Background literature

Loneliness has been defined as “subjective feelings of social isolation” (Hawkley and Cacioppo, 2010). By its very nature, loneliness is a unique and intensive unpleasant emotional experience characterized by social connection deficits. The current literature has conceptualized this construct in two main ways (Sinha and Wang, 2013). The first approach views loneliness as a unidimensional construct that remains constant across various contexts (Russell, 1996). The other conceptualizes loneliness as a multidimensional construct composed of two main subdimensions [i.e., social loneliness (SL) and emotional loneliness (EL)] (DiTommaso and Spinner, 1997; Heinrich and Gullone, 2006). EL results from deficits in emotional attachment to significantly intimate others (e.g., family members, romantic partners), whereas SL derives from a shortage of desired social networks or groups (e.g., friends and colleagues) (Russell, 1996). At the heart of a set of socio-emotional states, loneliness has garnered increasing attention from academia and public authorities during the past two decades. Research on CL has a relatively short history (Kim et al., 2005; Hu and Jasper, 2006; Arpin et al., 2015). The literature has examined the influences of loneliness in consumption contexts, such as the type of purchase (Pagan, 2020; Yang et al., 2021), consumption behavior (Yang, 2016), social experience and interaction (Kim et al., 2005; Her and Seo, 2018). Previous studies have also explored the role of loneliness in consumer experiences (Loh et al., 2021), attitudes (Yii et al., 2020), affects (Chen et al., 2021), preferences (Reid and Reid, 2007), and behaviors (Arpin et al., 2015; Liu et al., 2020). Moreover, CL has been investigated as a mediator or moderator in explaining consumer behaviors (Berezan et al., 2020; Pade and Feurer, 2022). Studies on CL are driven by the idea that relationship deficits in consumers’ daily lives play important roles in their attitudes, cognitions, affects, and behaviors (Mead et al., 2010; Lee and Hyun, 2015). Thus, the marketing literature has recently begun to shed light on CLs and pay increasing attention to the distinctiveness of lonely consumers (Rippé et al., 2018; Berezan et al., 2020; Wang et al., 2021). However, work on CLs remains scattered among various research fields, thus providing an inadequate understanding of the role of loneliness in the consumer journey.

## 3. Methodology

According to the five-step approach (Denyer and Tranfield, 2009; Vizzoto et al., 2021), this study firstly identified focus research questions. Specifically, this review aimed to present the antecedents (i.e., what influences CL?) and consequences (i.e., what does CL produce?) of CLs, and their role in consumer behaviors (i.e., does CLs play mediator/moderator role?). Accordingly, the above four research questions guided the review process.

Second, we began with a bibliographic keyword search of four databases: the Social Science Citation Index, Springer, ScienceDirect, and Google Scholar. These databases were chosen because they not only include service and marketing journals but also cover management, psychology, and information technology journals that have published CL studies. Several relevant keywords, such as consumer/customer loneliness and lonely consumer/customer, were used. During the process, loneliness studies that investigated or involved consumption behaviors were included in the review pool. Additionally, we applied backward and forward snowball search techniques to ensure the completeness of the review (Vizzoto et al., 2021). After this step, a total of 127 articles were collected from the focal databases.

In the third step, we evaluated and selected studies based on two key criteria: relatedness to the topic and research quality (Vizzoto et al., 2021). To be included in the review, a paper first had to meet the relatedness criterion; that is, CL or loneliness in the consumption context was one of the key subjects in the article. Accordingly, 58 articles unrelated to CL were eliminated. Moreover, with regard to research quality, an included paper had to be written in English and published in a peer-reviewed academic journal. We also analyzed empirical studies based on their methods, the results, and conclusions (Vizzoto et al., 2021). One article with low clarity and coherence was excluded. Thus, our final sample consisted of 68 research articles.

**FIGURE 1:**
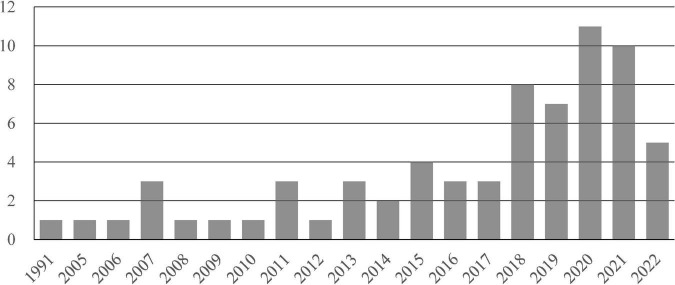
Number of articles between 1991 and 2022.

Fourth, the selected articles were analyzed by year, journal, methodology, research objective, and location. The articles covered the period from 1991 to 2022. Figure 1 details the distribution of CL studies by publication year. Although the number of publications in 2022 is incomplete, there has been an upward trend of publications over time.

Table 1 presents an overview of the literature on CL. The main sources for these works are *Journal of Business Research*, *Journal of Consumer Research*, and *Psychology and Marketing*. Of the identified articles, 12 are theoretical explorations and 56 are empirical works. Regarding their methodology, 24 articles have employed a cross-sectional survey method (42.9%), 18 studies have used an experimental design (32.1%), and 6 studies have used mixed methods (10.7%). With regard to their research objectives, 12 have examined the antecedents of CL, 27 have investigated the consequences of CL, and 2 articles have explored both of these. Moreover, six articles have examined the mediating role of CL, and five articles have explored the moderating effects of CL. Among these works, only four have been conducted in a cross-cultural context. Based on this literature overview, the next section reports and discusses the extant findings.

**TABLE 1:** Disciplines and publication outlets of consumer loneliness (CL) studies.

Category	Journal	Total	Empirical articles	Conceptual articles
Marketing and management	Journal of Business Research	5	5	0
	Journal of Consumer Research	5	5	0
	Psychology and Marketing	5	5	0
	Journal of Consumer Psychology	4	3	1
	European Journal of Marketing	3	3	0
	Journal of Marketing Research	2	2	0
	Australasian Marketing Journal	1	1	0
	International Journal of Consumer Studies	1	1	0
	International Journal of Retail and Distribution Management	1	1	0
	Journal of Business Ethics	1	1	0
	Journal of Consumer Behavior	1	1	0
	Journal of Retailing	1	1	0
	Journal of the Academy of Marketing Science	1	0	1
	Marketing Intelligence and Planning	1	1	0
	MIT Sloan Management Review	1	0	1
Service, tourism, and hospitality	International Journal of Hospitality Management	3	3	0
	Current Issues in Tourism	2	1	1
	Journal of Service Management	2	0	2
	Tourism Management	2	2	0
	International Journal of Contemporary Hospitality Management	1	1	0
	Journal of Retailing and Consumer Services	1	0	1
	Journal of Services Marketing	1	1	0
Information technology	Computers in Human Behavior	4	3	1
	Cyberpsychology and Behavior	3	3	0
	Cyberpsychology, Behavior, and Social Networking	3	3	0
	Journal of Management Information Systems	1	1	0
	Telematics and Informatics	1	1	0
Psychology	Current Opinion in Psychology	1	0	1
	Frontiers in Psychology	1	1	0
	Journal of Experimental Social Psychology	1	1	0
	Personality and Social Psychology Bulletin	1	1	0
	Psychological Reports	1	1	0
	Trends in Psychology	1	1	0
Others	British Journal of General Practice	1	1	0
	Frontiers in Public Health	1	0	1
	Pediatric Obesity	1	1	0
	The Social Science Journal	1	1	0
	Virtual Reality	1	0	1

## 4. Findings

### 4.1. Antecedents: What influences consumer loneliness?

#### 4.1.1. Predictors of consumer loneliness

From the social change perspective, the number of lonely consumers is increasing, potentially due to longer life spans, disconnected social networks, increasing social isolation, and single-person households (Wang et al., 2012). As a psychological experience, CL is exacerbated by changing socioenvironmental factors (Grigoropoulos and Daoultzis, 2022).

##### 4.1.1.1. The aging population

In 2050, the proportion of the world’s population aged over 60 years will be close to 22% (World Health Organization [WHO], 2021). Consequently, older consumers represent a rapidly growing segment in the global marketplace. Previous evidence has indicated that decreased social interaction increases older consumers’ loneliness (Kim et al., 2005). In addition, a lack of shopping mobility also contributes to older consumers’ loneliness (Lim and Kim, 2011). Due to their lack of social contacts, older consumers have fewer ways to resolve their loneliness. However, older consumers’ social needs have not been completely met in the marketplace and adequately understood by academics.

##### 4.1.1.2. Decreased social connectedness

Threats to valued social relationships, ranging from social exclusion to divorce, have been found to elevate CL (Jiao and Wang, 2018). Preliminary research has shown that affiliation deficits result in CL (Lastovicka and Sirianni, 2011). In addition, a meta-analysis of social media has indicated that a lack of social support results in loneliness (Song et al., 2014). Socially excluded consumers tend to devalue social relationships and thus experience greater loneliness (Mead et al., 2010). Similarly, a lack of psychological attachment to others has been identified as a significant antecedent of CL (Loh et al., 2021). Although prior studies have indicated that perceived solitude and social disconnectedness, rather than the actual amount of social contact, drive the sense of loneliness (Henkel et al., 2020), insufficient physical, and social activity play a role in facilitating CL (Han et al., 2022).

##### 4.1.1.3. Technology- and pandemic-induced alienation

The prevalence of CL has been accelerated by the development of information technologies and digitalized social networks (Yang et al., 2021). For example, Instagram broadcasting behaviors increase users’ loneliness (Yang, 2016). Moreover, social media users’ exchange relationship orientation enhances their perceived SL (Matook et al., 2015). After the outbreak of the COVID-19 pandemic, social distancing measures have been widely used, leading to even higher levels of social isolation. Prolonged lockdowns have further amplified the deleterious effects of the COVID-19 pandemic. Previous research has reported that the pandemic crisis increases older people’s loneliness (Wong et al., 2020). Furthermore, COVID-19-related fear increases CL and alcohol consumption (Grigoropoulos and Daoultzis, 2022).

##### 4.1.1.4. Consumer characteristics

Research using the personality approach has suggested that introverted consumers reduce their loneliness by interacting with service frontliners (Kim et al., 2005; Pettigrew, 2007; Rippé et al., 2018). Moreover, a meta-analysis has indicated that Facebook users’ shyness results in loneliness (Song et al., 2014). Insecure attachment has also been verified to increase EL (Rippé et al., 2021). In addition, consumers who engage in more consumer-ethnocentric activities (i.e., buying domestic products) feel lonelier (Yii et al., 2020). Demographic variables such as socioeconomic status, the quantity and quality of interpersonal interaction (Kim et al., 2005), age (Rokach and Neto, 2005), and gender (Salimi, 2011) have also been related to differences in the severity of CL.

##### 4.1.1.5. Culture and norms

Loneliness always emerges within the context of a culture with normative values and practices. Culture significantly affects the causes of loneliness (Rokach and Neto, 2005). For instance, individuals in collectivistic societies report higher loneliness than those in individualistic societies (Lykes and Kemmelmeier, 2013). Chinese cultural values are generally collectivistic and center on family orientation and kinship; thus, to address their loneliness, older Chinese consumers rely on interactions with family members to address their CL more than on social interactions with others in service or consumption settings (Song et al., 2018).

#### 4.1.2. Alleviators of consumer loneliness

##### 4.1.2.1. Consumption behaviors

The research has underscored that consumption is an effective tactic to ameliorate CL (Liu et al., 2020). Lonely consumers may consider acquiring possessions to show their status to others (Mandel et al., 2017). Although this “possession effect” recedes over time, it temporarily reduces CL (Pieters, 2013). Purchasing products that reflect a group affiliation (e.g., majority-endorsed sneakers) or consuming peer-recommended services (e.g., restaurants) helps achieve the assurance of consumers’ social self. Moreover, products/services that foster a sense of belongingness help consumers attenuate their loneliness perceptions (Yii et al., 2020). For instance, Pettigrew (2007) contended that consumption behaviors help senior consumers decrease loneliness while enhancing their affiliation. Recently, Wang et al. (2021) found that rituals in product consumption (e.g., eating Oreo cookies by following the twist-lick-dunk ritual) can reduce CL.

As a social experience, shopping is a feasible coping strategy that can play a key role in reducing CL (Smith et al., 2018). Lonely consumers go to retail stores and interact with salespeople to satisfy their need for social support (Rippé et al., 2018; Smith et al., 2018). For them, going shopping becomes a social activity to receive complementary social resources rather than an activity to acquire desired products and services (Kim et al., 2005). Thus, service establishments offer an ideal outlet for social activities and participation, thereby alleviating consumers’ perceived isolation and loneliness. Moreover, relative to material purchases, experiential purchases exert a greater impact on CL alleviation (Yang et al., 2021). For example, shopping malls have been identified as an important “third place” for lonely consumers to alleviate loneliness (Kim et al., 2005). In the restaurant service context, employees’ service manner exhibits a significantly positive effect on CL reduction (Song et al., 2018). Similarly, participation in consumption communities (Sullivan and Richardson, 2020) and holiday trips (Pagan, 2020) has been identified as effective alleviators of CL.

##### 4.1.2.2. Technology-facilitated connectedness

Currently, online social activities play a critical role in consumers’ daily lives. Adolescents use social media as a primary source of social relations to cope with pandemic-induced loneliness (Cauberghe et al., 2021). Uram and Skalski (2020) indicated that social media users’ life satisfaction significantly decreases their loneliness. Moreover, technology use (e.g., e-mail, social networks) predicts lower loneliness and benefits older consumers’ mental and physical health (Chopik, 2016). Virtual interactions on social media (e.g., Facebook) help users achieve interactivity, develop social skills, and facilitate a sense of belongingness (Berezan et al., 2020), thus combating their loneliness. Yang (2016) reported that Instagram browsing decreases users’ loneliness, while Instagram interaction exhibits a similar effect only on users with a low social comparison orientation. Moreover, in contrast to text-based social media (e.g., Twitter), image-based social media (e.g., Instagram) have a greater therapeutic effect on CL (Pittman and Reich, 2016). Even sounds in mobile apps can satisfy lonely consumers’ needs for belonging by creating a sense of social presence (Poupis et al., 2021).

### 4.2. Outcomes: What does consumer loneliness produce?

#### 4.2.1. Consumer behaviors

As a signal that individuals’ perceived social connections are not at their desired levels, loneliness spurs people’s efforts to escape from and change this painful state (Cacioppo et al., 2006). Thus, when consumers are plagued by loneliness, they go to great lengths to fulfill their need for others (Heinrich and Gullone, 2006).

##### 4.2.1.1. Shopping as a social experience

By considering shopping a social activity, lonely consumers can form personal bonds with others in the marketplace as substitutes for other social contacts (Rippé et al., 2018). These social contacts help consumers forge social connections (Lastovicka and Sirianni, 2011; Wang et al., 2021), fill their social voids, and blend in rather than stand out (Mead et al., 2010). Therefore, CL enhances not only consumption-oriented motivations (e.g., dining out) but also experiential-oriented motivations (e.g., traveling) (Lim and Kim, 2011). Furthermore, shopping-related experiences facilitate social reconnection and affiliation (Mandel et al., 2017). Lonely consumers tend to consider new sources of social relationships in an optimistic way and are eager to interact with others in consumption contexts (Liu et al., 2020). They purchase more domestic-choice products, which can affirm their social identity (Yii et al., 2020). Furthermore, spending and purchasing are strategically utilized by lonely consumers as affiliation tools in the service of forging social connections (Mead et al., 2010). Finally, consumption functions as a form of compensation. Compensatory consumption refers to “the desire for, purchase, or use of products/services to satisfy a psychological need” (Loh et al., 2021) and has been used by people feeling lonely to establish a sense of social connectedness (Mead et al., 2010; Yan and Sengupta, 2020). The therapeutic value of compensatory consumption has also been established (Kim and Jang, 2017). More specifically, consumers feeling lonely participate in compensatory consumption to fulfill their needs for belonging, affiliation, and self-esteem (Mead et al., 2010; Lim and Kim, 2011; Mandel et al., 2017).

##### 4.2.1.2. Online social activities

According to the social compensation model (Sirola et al., 2019) and the social augmentation hypothesis (Song et al., 2014), loneliness motivates consumers to engage in online social activities to enhance their social world. Online social resources (e.g., social networking sites, social media platforms) are often readily available, representing an accessible medium to compensate for relationship deficits. Lonely consumers spend more time web surfing and on social networking platforms as a substitute for real interpersonal interactions (Berezan et al., 2020). They benefit more from social applications, such as Facebook, than non-lonely consumers do (Song et al., 2014). Moreover, the use of online travel communities for social and emotional loneliness (OTS-SEL) stimulates users’ peer communication in online communities (Lee and Hyun, 2015). In tourism contexts, social CLs positively influence tourists’ smartphone application use (Tan and Lu, 2019). In addition, tourists may engage in online peer-to-peer accommodations (e.g., Airbnb) to escape loneliness (Farmaki and Stergiou, 2019).

##### 4.2.1.3. Parasocial interaction

Lonely consumers seek out parasocial relationships with media personalities, pets, or commercial relationships in “the third place” (Song et al., 2018). For instance, loneliness increases older consumers’ parasocial interactions (Lim and Kim, 2011). CL also facilitates consumers’ efforts to develop a sense of connectedness with non-human agents (e.g., pets, products, brands) (Liu et al., 2022). Lonely consumers tend to humanize brands and products/services (Dalman et al., 2021). CL may be a key driver of anthropomorphism in online and offline consumption contexts (Chen et al., 2017). A recent study on celebrity-following behaviors demonstrated that CL predicts followers’ parasocial interactions with celebrities’ social media (i.e., visiting frequency and following behaviors) (Kim et al., 2019).

##### 4.2.1.4. Impulsive and self-focused consumption

Loneliness impairs self-regulation, logical reasoning, and time perception (Wang et al., 2021). Thwarted belongingness makes consumers consume, even in personally unfavorable ways, for social affiliation (Mead et al., 2010). Thus, CL has been associated with impulsive, unhealthy, and problematic consumption (Atalay and Meloy, 2011; Rippé et al., 2021). Mead et al. (2010) contended that socially excluded consumers may exhibit distinct spending and consumption strategies. Consumers who experience essential relationship deficits, relative to consumers who experience inessential relationship deficits, engage more often in impulsive consumption (Sinha and Wang, 2013). Moreover, CL increases solitary consumption (e.g., alcohol consumption) while decreasing social consumption (spending time with others) (Arpin et al., 2015). Loneliness-induced coping strategies are significantly associated with adolescents’ unethical consumption behaviors (e.g., underage smoking) (Gentina et al., 2018). In addition, CL signals a priority for self-focused or self-oriented consumption (e.g., self-gifting) for hedonic rewards or a *carpe diem* mentality (Rippé et al., 2018; Fumagalli et al., 2022). Hence, loneliness has been considered the most significant predictor of problematic internet use (Ceyhan and Ceyhan, 2008), conspicuous consumption (Liu et al., 2020), and the consumption of sugar-containing foods and beverages (Doan et al., 2022).

#### 4.2.2. Consumer emotions

Loneliness influences the entirety of consumers’ daily lives (Rippé et al., 2021). Hence, CL has been linked to different consumer emotions. CL encourages consumers to reflect on and become attached to product/service consumption that represents their social ties (Lim and Kim, 2011; Mittal and Silvera, 2018). As a threat to one’s social self, CL triggers consumers’ purchase attachment tendency in terms of reaffirming social relationships. More specifically, lonely female (male) consumers become attached to material (experiential) purchases (Mittal and Silvera, 2018). Lonely consumers attach nostalgia to socially important purchases to bridge a perceived gap between their ideal and actual social bonds (Mittal and Silvera, 2018), yielding the perception of social connection (Loh et al., 2021). With regard to positive consumer outcomes, CL exerts a positive effect on consumer enjoyment in online social media (Kim et al., 2019) and offline retailing (Smith et al., 2018). Thus, satisfaction for lonely consumers stems from not only consumption itself but also the consumption experience (Kim et al., 2005). CL has also been related to negative consumer emotions. For example, loneliness devastates consumers’ wellbeing (Berezan et al., 2020). SL accelerates tourists’ leisure boredom at destinations (Tan and Lu, 2019). Furthermore, vulnerable consumers (e.g., consumers in late adolescence) are more heavily impacted by loneliness (Pagan, 2020; Loh et al., 2021). Among them, loneliness has been associated with several psychological issues (Sirola et al., 2019). Relative to anxiety, loneliness exhibited a greater impact on feelings of happiness during COVID-19 lockdowns (Cauberghe et al., 2021).

#### 4.2.3. Consumer preferences

Lonely consumers perceive themselves as different from others (Wang et al., 2012) and exhibit different consumption orientations and expenditure patterns (Kim et al., 2005). For instance, they prefer talking on cell phones and consider texting as a less intimate method of contact (Reid and Reid, 2007). Moreover, they prefer online social interactions for greater control over their self-presentation and anonymity. They demonstrate a preference for material consumption, even though it ultimately aggravates their loneliness (Pieters, 2013). Lonely consumers prefer domestic choices in food consumption settings (Yii et al., 2020). Moreover, they prefer minority-endorsed products that present a natural fit with their loneliness in private consumption contexts, but conform to majority-endorsed products to display their affiliation in public consumption contexts. Researchers have also found that lonely consumers prefer angular shapes over circular shapes only in private consumption contexts (Chen et al., 2021) and prefer products/services characterized as “warm” more those characterized as “cold” to satisfy their need for social connectivity (Chen et al., 2017). In addition, temporary CL makes consumers prefer various objects (e.g., products and prices) accompanied by divisible numbers (Yan and Sengupta, 2020).

#### 4.2.4. Consumer attitudes

For lonely consumers, a common coping strategy to relieve their loneliness is embracing materialism (Pieters, 2013; Gentina et al., 2018; Loh et al., 2021). EL increases consumer materialism (Rippé et al., 2021), which further enhances self-brand connections (Loh et al., 2021). However, loneliness and materialism reciprocally influence each other over time (Pieters, 2013). Although socially excluded consumers attach more importance to their possessions while experiencing greater loneliness (Mead et al., 2010), increasing evidence supports the phenomenon that CL contributes to materialism by crowding out social relationships (Pieters, 2013; Fumagalli et al., 2022). Moreover, the predictive role of CL in consumer ethnocentrism has also been established (Yii et al., 2020). In addition, lonely consumers report a negative attitude toward depersonalized retailing and service experiences (Kim et al., 2005; Hu and Jasper, 2006) and evaluate them more negatively than non-lonely consumers do (Forman and Sriram, 1991).

#### 4.2.5. Cognition and cognitive assessment

Consumers allocate more attention to social cues in consumption environments when they are lonely (Mead et al., 2010; Liu et al., 2020). Hence, scholars have examined the relationship between CL and product evaluation (Wang et al., 2012). For example, OTS-SEL increases users’ identification with peer groups (Lee and Hyun, 2015). Moreover, lonely female (male) consumers perceive material (experiential) purchases to have higher social value than experiential (material) purchases (Mittal and Silvera, 2018). In addition, SL positively influences tourists’ esthetic scope during their trips (Tan and Lu, 2019).

### 4.3. Consumer loneliness as a mediator

Loneliness helps explain the relationship between consumers’ interpersonal relationship deficits and possessive love (Lastovicka and Sirianni, 2011). Moreover, consumers who encounter group consumers (rather than mostly solo consumers) in restaurants experience greater loneliness, undermining their solo dining intentions (Her and Seo, 2018). Scholars have also shown that EL intervenes with the positive effect of insecure attachment on consumer self-gifting motivations (Rippé et al., 2021). In addition, Pade and Feurer (2022) identified CL as a mediator linking disadvantageous personalized pricing and perceived price fairness. CL also transmits the positive effect of the fear of COVID-19 on alcohol consumption behavior (Grigoropoulos and Daoultzis, 2022). When loneliness is considered a negative mediator, reduced loneliness mediates the relationship between technology use and physical and mental health among older consumers (Chopik, 2016).

### 4.4. Customer loneliness as a moderator

When modeled as a moderator, CL has been found to enhance the relationship between excessive gambling and participation in online gambling communities among adolescents and young adults (Sirola et al., 2019). CL also promotes the positive effect of affectionate gestures (e.g., hugging) on attitudes toward a target product imbued with anthropomorphic traits (Hadi and Valenzuela, 2014). Moreover, for Facebook users with high loneliness, psychological needs (e.g., belonging) contribute more to their Facebook usage than to the usage of those with low loneliness (Berezan et al., 2020). When product failure occurs, lonely consumers (relative to less lonely consumers) make fewer negative judgments about the focal brand (Dalman et al., 2021). In addition, empathy exhibits a significantly positive effect on moral identity and subsequent moral behaviors only among consumers who report greater loneliness (Jiao and Wang, 2018).

## 5. Future research agenda and implications for theory

### 5.1. Profiling lonely consumers

Generally, research on the antecedents of CL is relatively limited. There is little knowledge of loneliness-provoking factors in the consumption context. Although previous works have explored the role of demographic characteristics in CL, their findings have remained inconclusive. For example, some researchers have reported a non-significant gender effect, while others have not (Fumagalli et al., 2022). More attempts should be made to examine demographic effects and provide a clear profile of lonely consumers (Loh et al., 2021). Moreover, additional consumer traits should be integrated to explore the antecedents of CL (Yang et al., 2021). Consumer predispositions such as self-efficacy, innovativeness, empathy, and ethical identity should be examined as precedents of CL (Heinrich and Gullone, 2006; Mandel et al., 2017). This topic is of particular importance because personal traits interact with consumers’ feelings and often mold their behaviors (Li and Huang, 2022).

Cultural values usually set different baselines for the fulfillment of social needs while shaping consumer reactions (Song et al., 2018). For example, family interactions may be important for people from collectivistic societies to counteract loneliness, while interactions with friends are important for people in individualistic societies to alleviate loneliness (Lykes and Kemmelmeier, 2013). Thus, culture and social norms influence how CL is interpreted (Sirola et al., 2019). However, some researchers have found no significant group difference (e.g., between the U.S. and India) in CL-induced consumer reactions (Rippé et al., 2021). That is, the role of culture-related factors in loneliness has not yet been adequately understood. Cultural differences, in terms of power distance, uncertainty avoidance, or long-term orientation, have not yet been employed to explain the CL phenomenon (Rippé et al., 2021). Future research could more strongly address the relationship between cultural differences and CL and the effect of their interplay on consumer outcomes to present a picture of how consumers from different backgrounds feel loneliness.

### 5.2. Loneliness and consumer outcomes

#### 5.2.1. Quality of life

Quality of life (QOL) research is gaining momentum in the marketing and management literature (Sirgy et al., 2007; Li et al., 2022). QOL marketing refers to practices developed to enhance the wellbeing of consumers as well as firms’ other stakeholders (Li et al., 2022). Previous research has indicated that loneliness is associated with life satisfaction (Heinrich and Gullone, 2006). However, evidence supporting the relationship between CL and QOL is still lacking. The evolving service-dominant (S-D) logic suggests that all exchanges in the marketplace aim to improve the wellbeing of all stakeholders (Vargo and Lusch, 2017). Future studies should examine whether QOL marketing practices that aim to improve consumer welfare help to alleviate CL. This study also opens new areas for transformative service research (TSR) (Henkel et al., 2020). TSR centers on research that seeks to create uplifting changes in the wellbeing of all service entities. Lonely consumers tend to engage in more mood-regulation activities to repair their negative moods that accompany loneliness (Atalay and Meloy, 2011). Hence, researchers could explore the link between CLs and transformative services and their integrative effects on consumer outcomes. The marketing literature emphasizes positive long-term customer-firm relationships (Li and Huang, 2022). However, the literature offers little knowledge of the linkage between CL and customer-firm relationship (Lee and Hyun, 2015; Loh et al., 2021). For example, an increasing number of firms have adopted contactless services to mitigate the impact of the COVID-19 pandemic (Li and Huang, 2022). However, these practices decrease in-person interactions between customers and service providers, which are emphasized in traditional relationship marketing. Lonely consumers are sensitive to a depersonalized service environment (Hu and Jasper, 2006). Researchers could further explore the potential effect of CL in the relationship between technology-enabled services (e.g., AI-powered services) and customer satisfaction. In terms of the customer-firm relationship, the distinction between SEL needs to be further addressed (Heinrich and Gullone, 2006). Belongingness arises when an individual has frequent interactions with the same person in an enduring context of caring and concern (Baumeister and Leary, 1995). It is difficult for short-term social interactions to foster the close social relationships that emotionally lonely consumers need. That is, SL may be addressed by short-term consumption experiences and linked to positive consumer outcomes, while EL is more likely to be effectively alleviated by long-term customer-firm relationships. Furthermore, organizational behavior research has indicated that workplace loneliness weakens employees’ attachment and affective commitment toward their organization (Kloutsiniotis et al., 2022). Consumers are often viewed as “partial employees” in service firms (Li and Huang, 2022; Li et al., 2022). It is reasonable to postulate that the loneliness that emerges during the service process may frustrate consumers’ need for relatedness and undermine service relationships. In addition, because of the fear of loss and social anxiety (Baumeister and Leary, 1995), the relationship between service outcomes and customer loyalty may vary with the level of CL. Counterintuitively, a possible benefit of CL may be a stable customer-firm relationship. More specifically, when a firm develops loyalty among lonely consumers, those consumers will exhibit higher loyalty and associate more lifetime value with the firm than do non-lonely consumers. Hence, more work needs to be done to highlight the potential value of CLs in relationship marketing.

#### 5.2.2. Consumer behaviors

##### 5.2.2.1. Value co-creation behavior

Value co-creation has an inherently social nature; it facilitates a process that is conducive to promoting interactions and relationships among consumers and service providers (Li et al., 2022). Surprisingly, CL has rarely been related to customer value co-creation behaviors [i.e., customer participation, customer citizenship, customer-to-customer interaction (CCI), and customer-employee interaction]. The social motivations resulting from CLs may facilitate more service interactions during value co-creation (Li et al., 2022). Lonely consumers may engage in value co-creation as an outlet to seek social connectedness (Rippé et al., 2018). Furthermore, prior research has asserted that positive and lasting social relationships are important in helping people combat loneliness (Baumeister and Leary, 1995). Social interactions alone cannot help individuals combat loneliness, so the quality of the interactions should also be taken into account (Heinrich and Gullone, 2006). From this perspective, it is presumable that the effects of the co-creation experience on CL vary with service interaction quality (Li et al., 2022). Future research should focus on the links among CL, service experience, interaction quality, and other factors (e.g., S-D orientation) that enhance the therapeutic effects of value co-creation (Li et al., 2022). In addition, CCI has been found to significantly influence consumers’ social wellbeing (Altinay et al., 2019). Older consumers view fellow customers as a source of social contact and actively engage in CCI to combat loneliness. However, some studies have questioned the value of CCI (Nicholls and Gad Mohsen, 2015). The presence of other customers and lonely consumers’ anticipated evaluations also influence their choices and behaviors during public consumption (Wang et al., 2012). These mixed findings imply that there may be conditions under which the effect of CCI changes. For instance, introverted consumers may not benefit from interactions with unfamiliar fellow consumers because of their social anxiety (Her and Seo, 2018). In addition, loneliness has been related to a perceived lack of control over outcomes (Gierveld and Tilburg, 2006). Thus, lonely consumers are more likely to have an external service locus of control, which influences their value co-creation performance and service outcomes evaluation (Li et al., 2022).

##### 5.2.2.2. Consumption behavior

In terms of social functions, CL alerts consumers to engage in various consumption behaviors to alleviate loneliness (Fumagalli et al., 2022). CL has been operationalized as a chronic personality trait or a set of incident-induced temporary perceptions (Mead et al., 2010). However, the majority of CL research has focused on chronic loneliness, and very little is known about whether transient loneliness has similar effects in terms of consumer behaviors (Arpin et al., 2015). Thus, an interesting research question arises: do the social relationships established while shopping/purchasing help counteract chronic CL? If so, how long does this effect persist? Scholars should clearly articulate the definition of CL (as a situational or trait variable) in future studies and elaborate their differences. Moreover, whether and how different types of consumption reduce CL have yet to be examined (Song et al., 2018). For example, social interactions in retailing contexts may help reduce consumers’ SL but are less likely to alleviate their EL. Demonstrating the distinction between the different types of consumption is important for deepening our understanding of lonely consumers’ behaviors (Sinha and Wang, 2013). Another route for future research relates to the impacts of different types of CLs on consumption behaviors. Previous research has not distinguished SL from EL (Loh et al., 2021). Future research should consider CL as a multifaceted concept and investigate the effects of the type of loneliness on the service experience and prospective consumption. In addition, lonely consumers have a greater preference for services or products characterized by social attributes (Yang et al., 2021). However, not all social experiences are equally effective in reducing loneliness (Lykes and Kemmelmeier, 2013). Relative to material-based consumption, experience-based consumption leads to higher levels of social wellbeing (Kim and Jang, 2017). More specifically, experiential purchases (e.g., travel and sightseeing) can effectively enhance the social connections among consumers and others (Pagan, 2020; Yang et al., 2021). Moreover, hedonic consumption (e.g., leisure activities), relative to instrumental consumption (e.g., buying shampoo), may exert a greater influence on CL reduction. Limited research has examined the effects of specific consumption experiences on CL, providing an additional direction for future research.

##### 5.2.2.3. Consumer loneliness in the new era

The literature has paid little attention to how loneliness affects consumers and how consumers react in the new era (Sirola et al., 2019; Loh et al., 2021). For example, the development of intelligent technologies [e.g., service robots and intelligent personal assistants (IPAs)] facilitates the establishment of two-way parasocial relationships among consumers and service entities (Henkel et al., 2020). It has also been postulated that social robot services (e.g., communication and entertainment) have the potential to support lonely consumers (Henkel et al., 2020). Companion robots can ameliorate users’ loneliness through emotional support (Odekerken-Schröder et al., 2020). Therefore, service robots (e.g., Pepper by SoftBank) and IPAs (e.g., Amazon’s Echo Alexa) have the potential to offset the negative impacts of the pandemic while building rapport with consumers (Henkel et al., 2020). However, no empirical work has examined the above effects. Furthermore, intelligent technologies are increasingly considered emotional connectors, and the effects of social/emotional CL on consumer behavior toward intelligent technologies (and *vice versa*) should be investigated in emerging consumption contexts.

According to social surrogacy theory (Poupis et al., 2021), consumers seek out social surrogates to combat loneliness (e.g., pets, favored TV programs). Consumers’ identification with anthropomorphized objects helps facilitate a sense of connection (Loh et al., 2021). Odekerken-Schröder et al. (2020) proposed that intelligent technologies, from IPAs to humanized robots, can play three different roles (e.g., personal assistants, relational peers, and intimate buddies) in counteracting CLs. Thus, it is reasonable to suggest that each role offers specific social relations and contributes differently to CL alleviation, especially among adolescent consumers who are heavy users of smart digital devices and internet-based services. In addition, research has noted that online social networking cannot compensate for offline social deficits to adequately counteract loneliness (Sirola et al., 2019). The displacement hypothesis explains that time spent on a particular social medium (e.g., Facebook or online games) replaces time spent on other social mediums (e.g., family and friends) (Song et al., 2014). Heavy usage provides “leaner and less satisfying” social interactions, exacerbates social isolation, and worsens users’ psychological wellbeing (Yii et al., 2020). That is, CL may explain the dynamic link between changing social situations and social media behaviors. Future CL research could further clarify the paradox of social networking and the conditions under which these relationships vary.

Impacted by the coronavirus, human service delivery has been considered harmful and even lethal to both sides of the service dyad (Henkel et al., 2020). Service industries have had to adopt more technology-enabled services (e.g., contactless service) to increase their business. However, these pandemic-induced consumer experiences may also evoke CL (Rippé et al., 2021). The elevated loneliness caused by the pandemic quarantine may amplify and intensify vulnerable consumers’ negative psychological reactions to the pandemic crisis (Grigoropoulos and Daoultzis, 2022). Even amid the easing of pandemic-induced service measures, lonely consumers may continue to live under restricted social contact and suffer negative psychological outcomes (Henkel et al., 2020; Li and Huang, 2022). Thus, understanding what and how service practices are effective in alleviating CL during, and even after, the COVID-19 pandemic is essential to the service literature.

##### 5.2.2.4. Consumer loneliness changes over time

Loneliness can be a fleeting mood or a persistent experience that changes over time (Berezan et al., 2020). It motivates consumers to purchase products/services to achieve social connectivity (Mead et al., 2010; Pieters, 2013). However, consumption practices have been found to decrease while increasing CL. For example, shopping can be a coping mechanism to mitigate CL, even if only temporarily (Forman and Sriram, 1991; Rippé et al., 2018). Consumers can cheer themselves up by self-gifting, which has been proven to reduce negative moods while reinforcing positive moods (Rippé et al., 2021). Short-term consumption behaviors can assuage CL while enhancing consumers’ perceptions of social connection (Fumagalli et al., 2022), but a long-term reliance on consumption may undermine the high-quality social resources that are essential to dispelling loneliness (Sinha and Wang, 2013). A basic premise underlying CL studies is that the sense of loneliness motivates consumer behaviors that aim to alleviate it. Thus, loneliness should first be considered an antecedent of consumption behaviors and then be cured. More empirical attempts should further examine the reciprocal relationships between consumer behaviors and CL over time.

### 5.3. Implications for the theoretical lens and methodology

#### 5.3.1. Theories to explain the phenomenon of CL

Different theoretical lenses have been employed to explain the CL phenomenon, including the need to belong theory (Mead et al., 2010; Kim and Jang, 2017), theory of attachment (Gentina et al., 2018; Rippé et al., 2021; Pade and Feurer, 2022), and self-discrepancy theory (Mandel et al., 2017). Further work on CL could leverage alternative theoretical frameworks to capture the dynamic and multidimensional conceptualization of CL. Positive psychology research focuses on pursuing QOL *via* positive subjective experiences, which may provide new perspectives (Seligman, 2004). According to self-determination theory (Li et al., 2022), lonely consumers tend to seek substitutes when their basic needs for relatedness are frustrated. Social support, connectedness, and relatedness are vital to forming consumers’ wellbeing (Berezan et al., 2020). Similar to social baseline theory (Doan et al., 2022), social connections with others are not only an essential characteristic but also an important innate need of consumers. Lonely consumers tend to seek various substitutes when their basic need for relatedness is frustrated. Companion service robots have the potential to ameliorate CL *via* perceived social connections (Odekerken-Schröder et al., 2020). Following social exchange theory, trust and support from other beings can combat the negative influence of CL. Future research could examine the potential therapeutic effects of robotic services on CL across more contexts (e.g., restaurants and hotels) *via* these perspectives. In addition, the absence of meaningful interactions could predict CL perceptions (Grigoropoulos and Daoultzis, 2022). Relational theory suggests that healthy psychological development is facilitated by the context of authentic relational connection and mutual affirmation (Rippé et al., 2018), providing new insights for understanding CL.

#### 5.3.2. Research design for consumer loneliness studies

With regard to the conceptualization of CL, only four of the reviewed works use the term “CL.” The majority of the body of CL literature uses the more general terms “loneliness.” It is critical to further clarify the conceptualization of CL, especially for empirical studies. With regard to operationalization, CL has been measured in a variety of different ways and in various settings. For studies employing an experimental design, CL can be manipulated as a temporary emotional state (Cacioppo et al., 2006). Given the difficulty and possible ethical issues of the experimental manipulation of loneliness, much of the research on CL has used cross-sectional methods. For studies using the survey method, CL is measured by scales, such as the UCLA Loneliness Scale (Russell, 1996), the social, family and romantic loneliness scale (DiTommaso and Spinner, 1997), the De Jong Gierveld Loneliness Scale (Gierveld and Tilburg, 2006), and the three-item overall loneliness scale (Hughes et al., 2004). One exception is the OTS-SEL scale developed in online community settings (Lee and Hyun, 2015). Although previous works have yielded theoretically meaningful findings, we call for more domain-specific measures to assess CL accurately. Establishing a reliable, valid, and parsimonious measurement of CL can help the consumer literature obtain fruitful findings in the near future.

This study also calls for more research designs in future CL research. Several works have shown a reciprocal process of CL and consumer outcomes (Pieters, 2013; Yii et al., 2020). However, the methodologies used in previous studies have difficulty capturing the fluctuating experience of loneliness (Arpin et al., 2015). Thus, longitudinal designs should be introduced in CL studies, especially in those exploring chronic CL predictors and impacts on consumer outcomes. Moreover, CL fluctuates daily and over time. Hence, the diary methodology, which can help capture a consumer’s life as “it is lived,” can precisely delineate the intrapersonal variation in CL (Arpin et al., 2015). In addition, because consumers increasingly rely on online social resources (e.g., social media and online communities), emerging techniques (e.g., data mining and web mining) can also be employed to detect variations in CL and provide compelling results (Aria and Cuccurullo, 2017; Correa et al., 2019; Teichert et al., 2020).

## 6. Implications for practice

As a growing segment, lonely consumers’ needs remain unfulfilled in the marketplace (Fumagalli et al., 2022). Although every consumer’s experience of loneliness is unique, CL is not an untreatable condition. Researchers in clinical psychology have observed four categories of responses when people suffer loneliness: active solitude, spending money, social contact, and sad passivity (Heinrich and Gullone, 2006; Arpin et al., 2015). Accordingly, we provide practical implications for practitioners.

### 6.1. Lonely consumers who choose active solitude

Currently, the emergence of solo consumers is a significant trend in the global marketplace. Education, occupation, and lifestyles continue to expedite the growth of solo consumption (e.g., solo travel, solo dinning). Cacioppo et al. (2014) have suggested that “solitude expresses the glory of being alone.” Evidence from psychology and consumer research has affirmed that consumers who are alone are not equivalent to lonely consumers. The former spend more time alone and prefer solitary or solo consumption. Because they voluntarily choose to be solo consumers, marketers should keep in mind that some consumers who enjoy solitude are not lonely (Wang et al., 2012). Thus, in terms of service provision, proactive service practices with these customers may not be suitable and may even result in refusal because they may enjoy their consumption process alone.

### 6.2. Lonely consumers who choose to spend money

Consumption behaviors have been deemed a useful means to mitigate CL (Yang et al., 2021). Accordingly, more products/services with specific social benefits (e.g., belongingness and affiliation) should be developed to cater to this segment. For these consumers, spending money is a way to achieve social connectedness while mitigating CL, but it is not the end of the story. Second, this review has indicated that experiential purchases precede material purchases in attempts to reduce CL. Thus, when marketers in service industries promote their offerings, more information on therapeutic experiences for CL should be taken into account. Third, firms should create an organizational climate that facilitates social interactions and contacts with customers. For example, service establishments (e.g., coffee shops, banks, and shopping malls) could design a hospitable experience to initiate these practices. By addressing consumers’ enjoyment of socializing and communicating with others, marketing activities can help them fulfill their social affiliation needs.

### 6.3. Lonely consumers who seek out social contacts

When firms encounter lonely consumers who seek out social contacts, the following practices are suggested. First, marketers should provide an ideal platform for them to initiate social contacts and facilitate a sense of belonging. Managerial practices that aim to facilitate social interactions should be adopted, such as customer participation strategies that engage lonely consumers in interactions to enhance social connectedness (Li et al., 2022). Second, firms should provide more avenues to interact with these consumers. Some mediums are designed to enhance social connectedness (e.g., social media and instant communication apps). Firms can capitalize on these online social resources to meet the social needs of lonely consumers. Moreover, marketing practices that aim to create and maintain positive connections with consumers should be developed and implemented to dispel CL, such as practices guided by the service climate and S-D logic. Value co-creation (co-design, co-producing, etc.) can also be a promising strategy to engage consumers in interactive activities. Third, it is worthwhile to consider whether social media alleviates CL as expected. Marketers should remain cognizant of the trade-off between the benefits facilitated by intelligent technologies and lonely consumers’ desire for in-person interactions. When service settings lack social stimulation and a lively atmosphere, CL occurs as a negative outcome.

### 6.4. Lonely consumers who choose sad passivity

Consumers who employ sad passivity (e.g., overeating and drinking alcohol) to counteract loneliness are more likely to be at risk for poorer psychological and physical health (Arpin et al., 2015). Therefore, QOL and consumer education programs (e.g., social skill training and social support networks) can be integrated into marketing practices. These practices cannot only satisfy these consumers’ social needs but also foster consolidated customer-firm relationships so that firms can benefit in the long run. Moreover, more practices aimed at enhancing belongingness should be implemented. Firms could develop consumer communities to establish relationships and identification among consumers, service providers, and other customers. Consumer communities have been considered a cost-effective marketing tactic to enhance consumer attachment while combating CL. In addition, social technologies could be adopted to supplement these consumers’ social resources. Social technologies (e.g., social robots) provide significant psychological benefits (e.g., companion, friendship, and social identity) to lonely consumers. They “interact with humans in socially meaningful ways,” helping these consumers deal with loneliness regularly and every day (Odekerken-Schröder et al., 2020).

## 7. Conclusion

The steadily increasing segment of lonely consumers is worthy of attention in its own right. However, no attempt has been made to provide a comprehensive picture of CL research. In this review, we demonstrate how CL, as an emerging research topic, has received increasing attention from academia and practice. By bringing together its disparate research findings and integrating the literature’s extant knowledge on CL, this study not only facilitates future research by offering some promising avenues but also provides several practical implications. Nevertheless, this study is subject to several limitations. Although the development of the CL literature is at the beginning stage, we encourage future studies to employ more cutting-edging review research techniques to present a more precise science map, as recommended by Correa et al. (2022). The restricted search keywords may have excluded some related works that were not directly related to CL. Future review studies should include additional studies that examine CL-related phenomena in more contexts. Moreover, this review emphasizes empirical evidence from CL research; thus, some insights from conceptual studies may not be fully articulated. By integrating some insightfully theoretical and anecdotal evidence, future studies could present a better understanding of the growing CL phenomenon.

## Data availability statement

The raw data supporting the conclusions of this article will be made available by the authors, without undue reservation.

## Author contributions

Both authors contributed equally to the work and approved it for publication.
